# Supplementary material for: Single nuclei and spatial profiling of sacrococcygeal teratomas reveals cellular composition and X inactivation heterogeneity
Source: NPJ Precis Oncol. 2026 Jan 21;10:87. doi: 10.1038/s41698-025-01262-4 (PMC12949016; doi:10.1038/s41698-025-01262-4)
Supplement: Supplementary file 1 — Supplementary Information [file 41698_2025_1262_MOESM1_ESM.pdf]

SUPPLEMENTARY MATERIALS FOR:

Integrated single-nuclei and spatial transcriptomic profiling of human sacrococcygeal teratomas reveals heterogeneity in cellular composition and X-chromosome inactivation

Ernesto J. Rojas[1,2,3,4,5], Krinio Giannikou[6], Benjamin J. Huang[4,7], Soo-Jin Cho[8], Marco Cordero[9], Deion Pena[10], Lan Vu[3,9], Aditya Bagrodia[6], S. Christopher Derderian[10], Tippi C. MacKenzie[3,5,9,*], Diana J. Laird[1,2,4,5,*]

Co-Corresponding authors: diana.laird@ucsf.edu & tippi.mackenzie@ucsf.edu

This PDF includes:
Supplementary Figs. 1 to 5

Other Supplementary Materials for this manuscript include the following:
Supplementary Tables 1 to 4 (Excel) provided separately.

Supplementary Figures

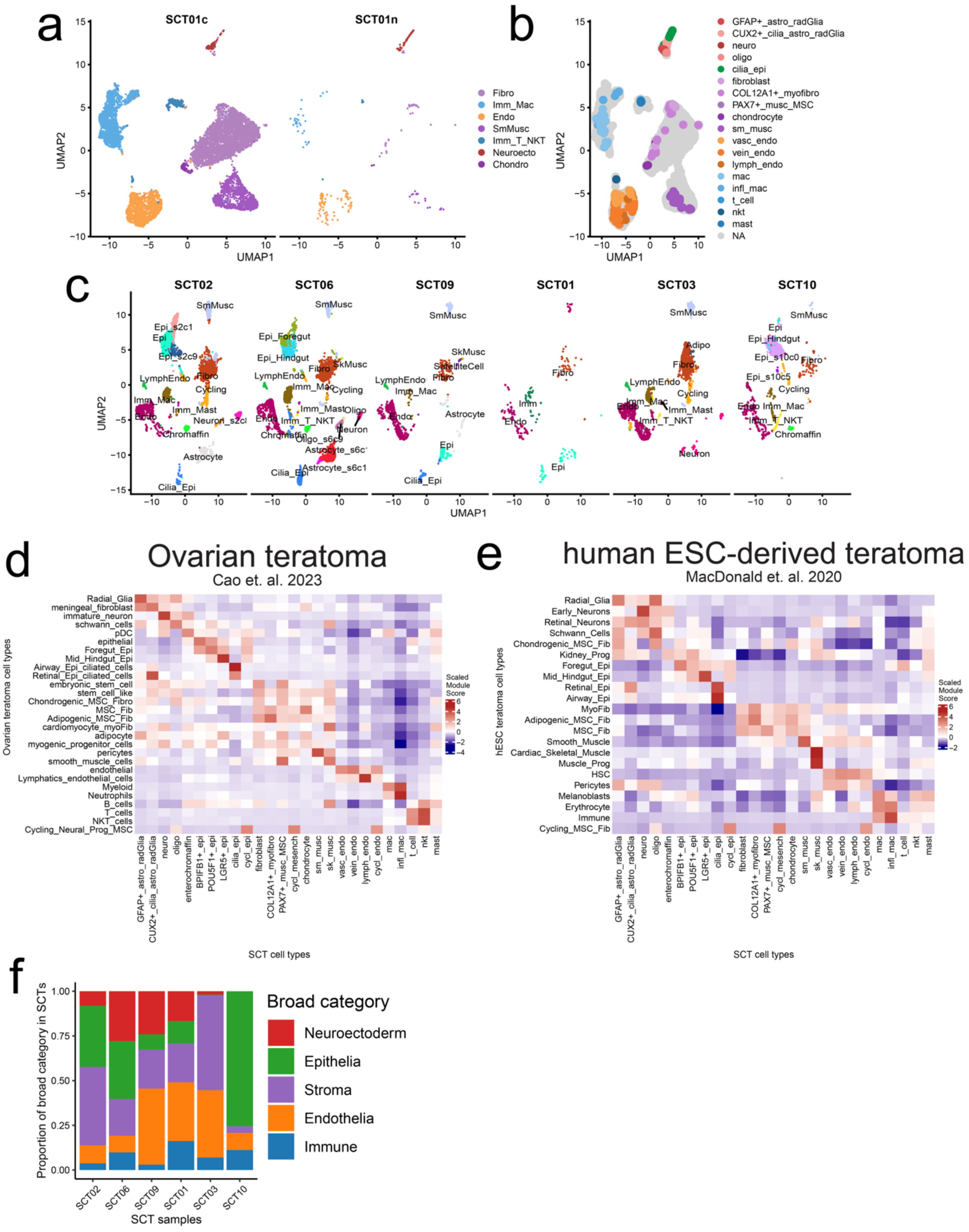

Supplementary Figure 1. Prior teratoma and developmental datasets informed annotation of cell types.

(a) UMAP projection of integrated single-cell (SCT01c) and single-nuclei (SCT01n) samples taken from SCT01. (b) Overlap/projection of SCT01n on SCT01c. For technical reasons, SCT01n was left in -80C rather than liquid nitrogen, leading to low cell capture. However, of the cells captured there was broad overlap in cell identities between cells and nuclei in SCT01. We proceeded with

nuclei for further analyses given nuclei captured all cell types independent of dissociation techniques. (c) Independent SCT annotations from each sample allowed for the naming of cell types among the individual tumors. We then mapped the cells from each cluster onto the same integrated UMAP space to validate overlapping identities. (d,e) Heatmap displaying the correlation between SCT sub-category clusters (x-axis) and ovarian teratoma clusters (y-axis) in d and human ESC-derived teratoma clusters (y-axis) in e. Red denotes high correlation and blue denotes low correlation. Most cell identities had considerable overlap, but there were some discrepancies in the names provided for the stromal population. (f) Bar plot colored by broad cell category from Figure 1b and grouped by SCT sample. SCT10 is the only sample missing a broad category - neuroectodermal lineage.

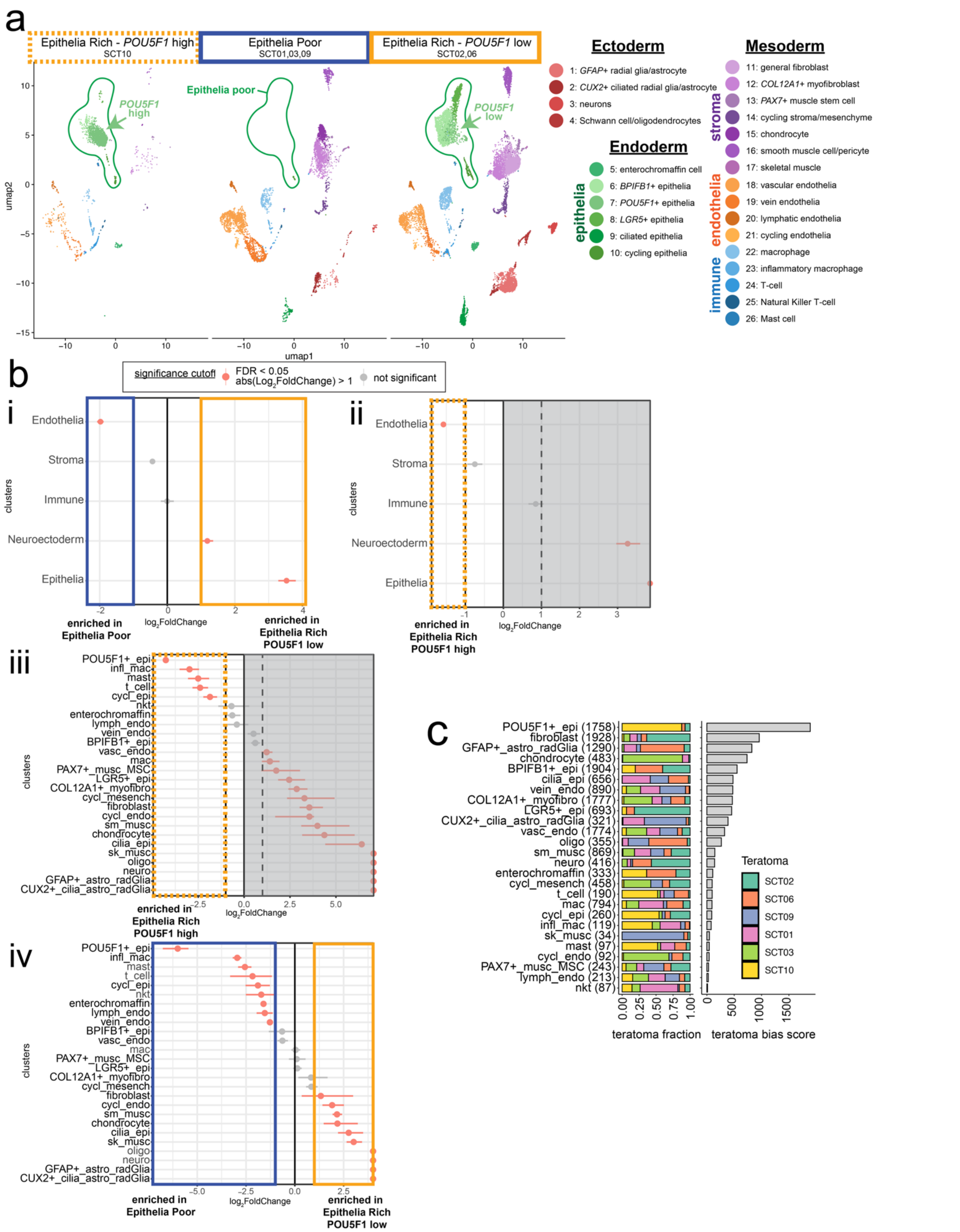

Supplementary Figure 2. Characterizing the proportional differences in cell types among the three SCT clusters.

(a) UMAP projection of the 3 categories of SCTs, (1) SCT10 - epithelia-rich *POU5F1*^{high}, (2) SCT01, 03, 09 - epithelia-poor, and (3) SCT02,06 - epithelia-rich *POU5F1*^{low}. Green arrow pointing to

the *POU5F1+* population that is enriched in SCT10 (epithelia-rich POU5F1high) and is present in lower proportion in epithelia-rich *POU5F1*^{low}. Green circle shows the missing epithelia population in epithelia-poor samples. (b) Proportion testing for cell type enrichment across the 3 principal component (PC) clusters. Orange dots are significantly enriched with false discovery rate < 0.05 and absolute log_2Fold Change > 1 by Monte-Carlo/permutation test. Boxes around the enriched results correspond to their PC cluster: dashed-orange box is epithelia-rich *POU5F1*^{high}; solid-blue box is epithelia-poor; solid-orange box is epithelia-rich *POU5F1*^{low}; greyed out area is the comparison for epithelia-rich *POU5F1*^{high}. (i) We show broad cell types for epithelia-rich *POU5F1*^{high} as compared to the rest of the samples and (ii) epithelia-poor as compared to epithelia-rich *POU5F1*^{low} SCTs. (iii-iv) The same comparisons for the sub-category cell types enriched in each of the SCT samples. (c) On the left: the proportional makeup of each sample across cell type, with total cell number in parentheses. On the right: the bias score for each cell type shows bias towards specific teratomas. A low bias score means the cell type is well mixed across all 7 teratomas, whereas a high bias score means that the identity is made up by mostly one or few SCT samples. The highest bias score was in *POU5F1+* epithelia and it was made up of 87% from SCT10, 8% from SCT02, 5% from SCT06, 0% from SCT01, 03, and 09.

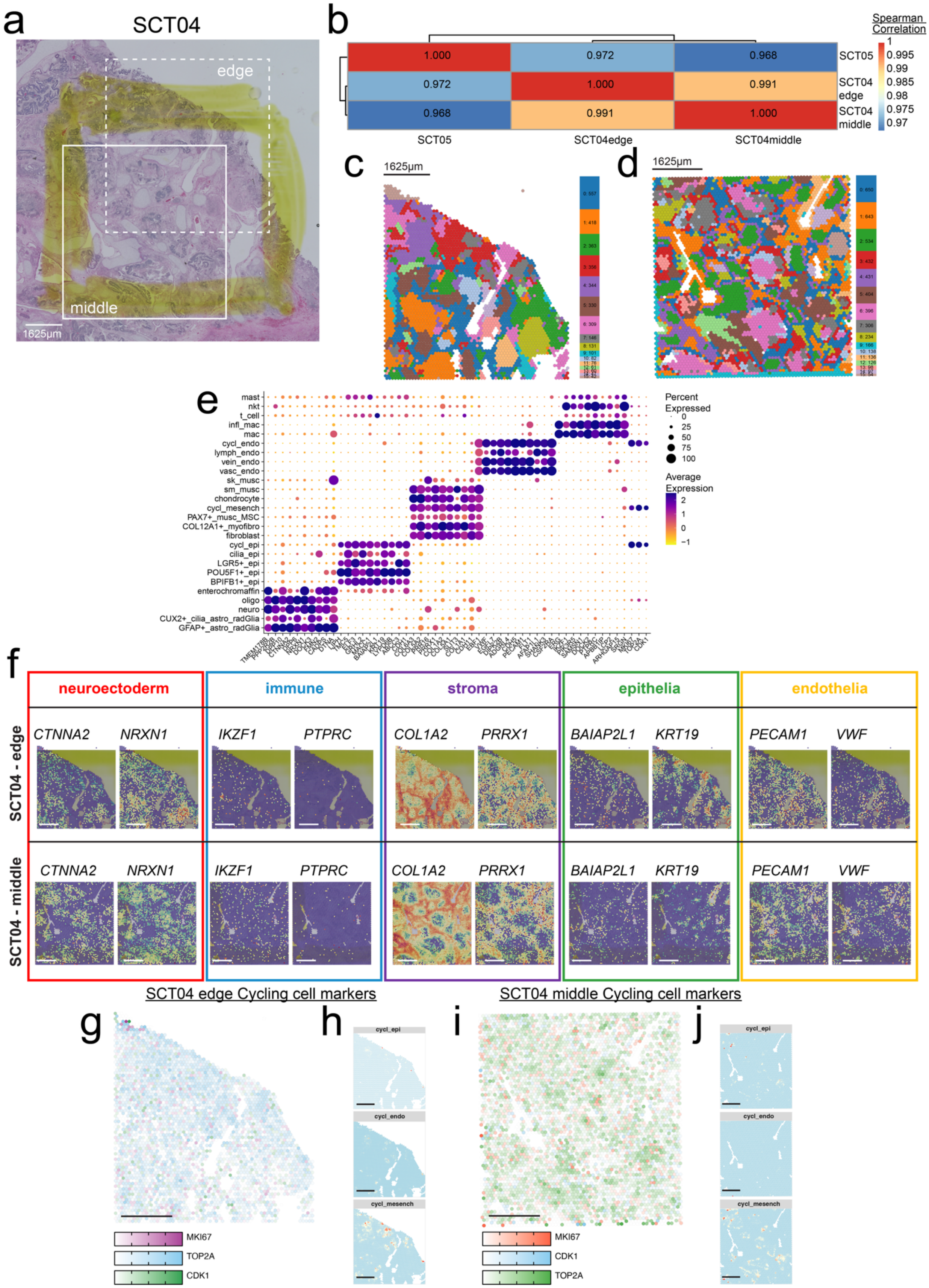

Supplementary Figure 3. Spatial transcriptomics of prenatal SCTs solid tumors.

(a) Hematoxylin and eosin staining of prenatally-resected SCT (prSCT), SCT04, with box showing what areas denote the “edge” and “middle” spatial datasets. (b) Spearman correlation reveals that the two spatial datasets from SCT04 correlate more strongly with each other than with

SCT05. Clustering was done by Euclidean distance. (c,d) Unbiased clusters overlaid in spatial plot for SCT04 edge (c) and middle (d). (e) Dot plot of key marker genes for each of the five broad categories of cell types in the snRNA-seq atlas. (f) Overlay of broad category markers taken from snRNA-seq atlas. Boxes correspond to the colors from Figure 1c, neuroectoderm in red, immune in blue, stroma in purple, epithelia in green, and endothelia in orange. (g,i) Cycling cell genes expressed across the SCT samples. Colors correspond to each gene: red = *MKI67*, green = *TOP2A*, blue = *CDK1*, with white being no expression. (h,j) Spatial distribution of cycling cell subtype spatial intensities by deconvolution analyses. All scale bars = 1625μm.

a

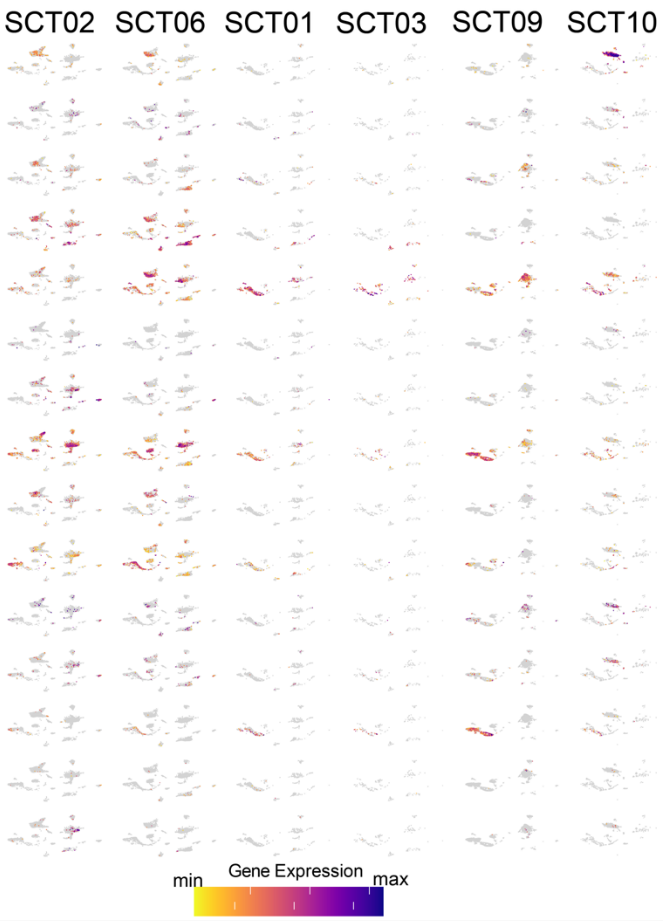

b

*Genes expr. above threshold	SCT02	SCT06	SCT09	SCT01	SCT03	SCT10	Total cells
None	5070	4579	803	199	2490	832	13973
DAZL	37	3	0	0	3	1	44
DDX4	2	3	0	0	4	0	9
NANOG	8	15	3	0	2	1	29
POU5F1	46	76	2	0	1	1152	1277
PRDM1	734	454	69	14	384	53	1708
SOX17	11	12	24	13	75	10	145
SOX2	167	368	26	25	7	0	593
TFAP2C	47	51	0	0	5	0	103
DAZL & PRDM1	6	0	0	0	0	0	6
DAZL & SOX2	1	2	0	0	0	0	3
DAZL & TFAP2C	1	0	0	0	0	0	1
DDX4 & POU5F1	0	0	0	0	0	1	1
DDX4 & PRDM1	1	0	0	0	0	0	1
DDX4 & SOX2	0	1	1	0	0	0	2
NANOG & POU5F1	1	0	0	0	0	1	2
NANOG & PRDM1	3	2	0	0	0	0	5
NANOG & SOX2	0	0	1	0	0	0	1
POU5F1 & PRDM1	2	0	1	0	0	5	8
POU5F1 & SOX2	0	3	0	0	0	0	3
POU5F1 & TFAP2C	4	3	0	0	0	3	10
PRDM1 & SOX17	1	2	8	2	59	5	77
PRDM1 & SOX2	12	6	0	0	0	0	18
PRDM1 & TFAP2C	7	0	0	0	0	0	7
SOX2 & TFAP2C	3	3	0	0	0	0	6
DAZL & POU5F1 & PRDM1	0	0	0	0	0	1	1
PRDM1 & SOX2 & TFAP2C	0	1	0	0	0	0	1

**Genes tested: POU5F1, NANOG, SOX2, SOX17, PRDM1, TFAP2C, DDX4, DAZL*

c

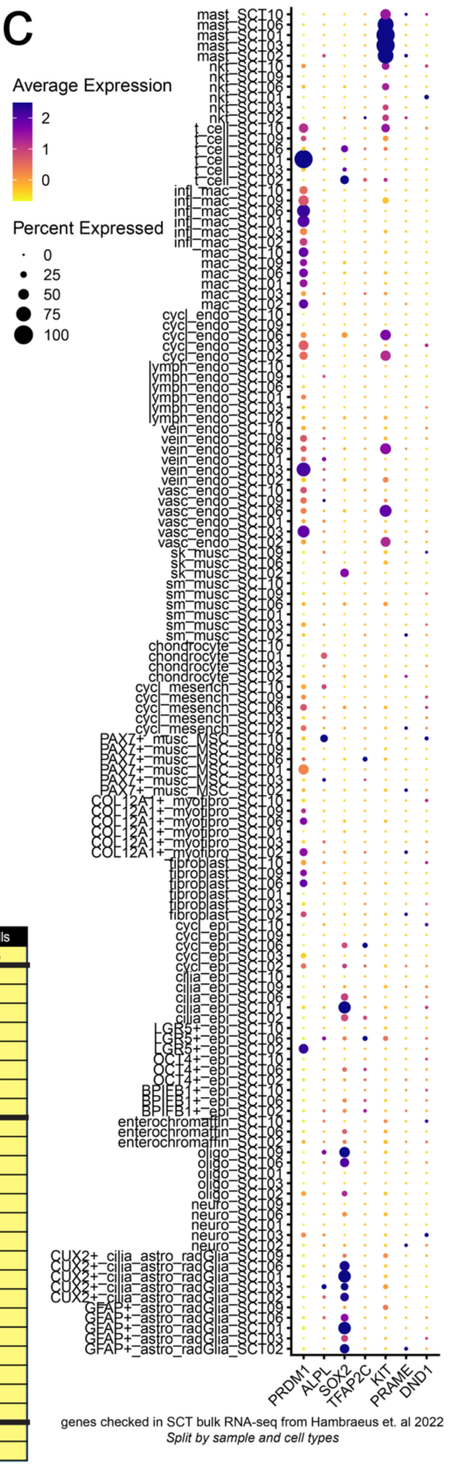

Supplementary Figure 4. Detailed mapping of genes found to be overexpressed in SCTs.

(a) Feature plot showing the expression of each of the pluripotent and PGC genes from Figure 4a. (b) Combinatorial expression of key pluripotent and PGC master regulators: *POU5F1, NANOG, SOX2, SOX17, PRDM1, TFAP2C, DDX4, DAZL*. Dark lines distinguish cells that express no combinations, single-positive, double-positive, and triple-positive cells. On the right highlighted in yellow is the total cells that express each combination above the threshold of normalized expression > 0.5. (c) Dot plot of genes that were described as expressed in bulk RNA-seq of SCTs expanded from Figure 4B split by sub-category cell types. Scale bars = 1625μm.

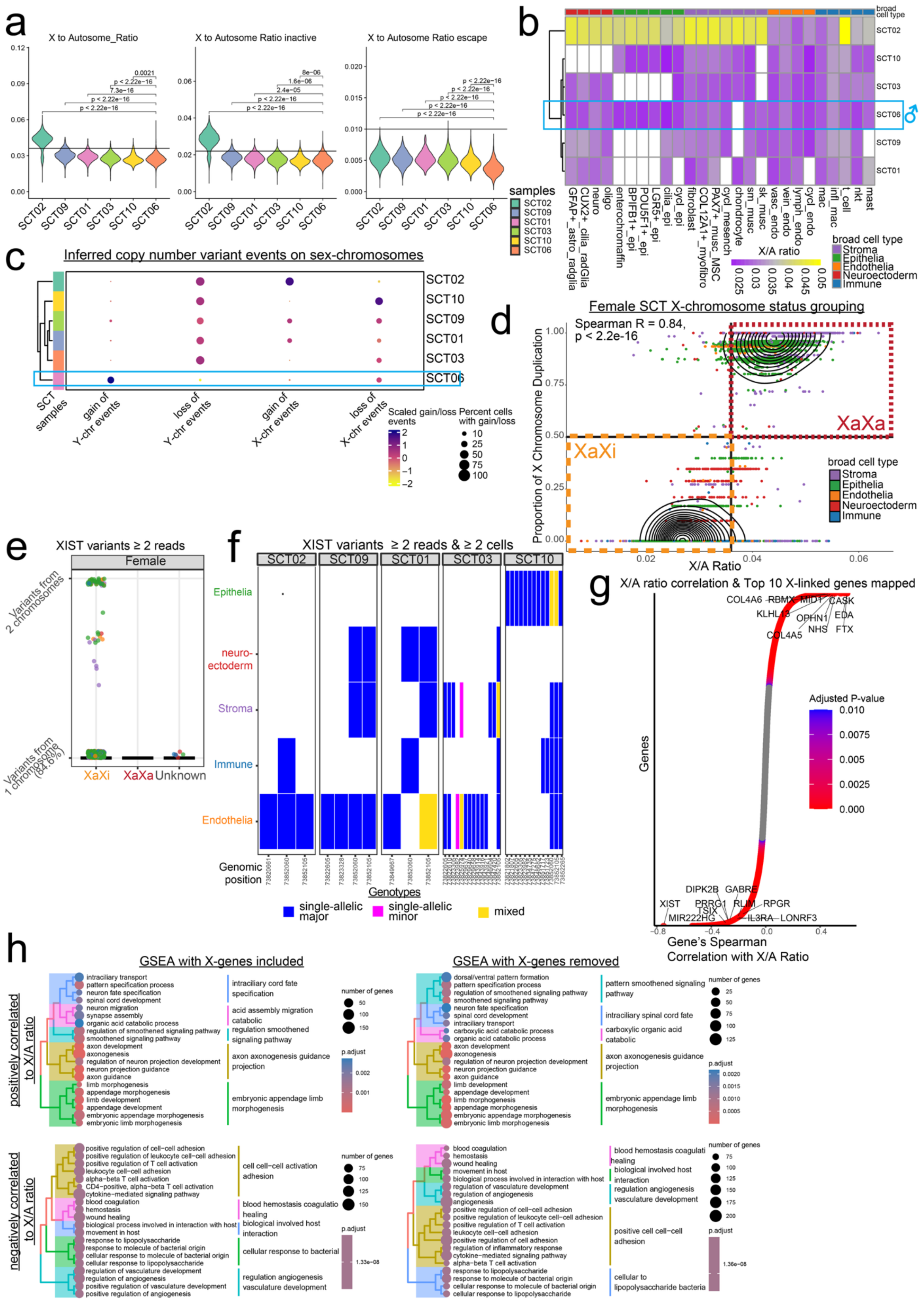

Supplementary Figure 5. Characterization of the X-chromosome gene expression for female SCTs.

(a) X-to-Autosome (X/A) ratio violin plots stratified by SCT samples. The three plots are then separated by overall X/A ratio, X/A ratio for X-linked genes that are inactivated during X inactivation

(XCI), and X/A ratio for X-linked genes that are escapees XCI. (b) Heatmap plot of the SCT samples (rows) and the sub-category cell types (columns). Purple shows low X/A ratio (< 0.036), yellow indicates high X/A ratio (> 0.036), with grey being 0.036. Broad cell types are highlighted at the top of the columns. (c) Dot plot showing inferred gain and loss events on the sex chromosomes from inferCNV with the male sample highlighted as successfully showing presence of Y-chromosome. (d) Scatter plot showing a significant positive correlation between the X/A ratio and the proportion of the X that is duplicated (R=0.84, p<0.01). We used a cutoff of X/A ratio > 0.036 and proportion of the X duplicated > 0.5 to call cells that we suspect have two active Xs (XaXa-like, burgundy box) versus those that have one active X (XaXi, orange box). Cells that did not land in these quadrants were designated as “unknown”. Density plots are overlaid to show where the cells are most concentrated. (e) Genotyping of single nucleotide polymorphisms (SNPs) on single-nuclei RNA molecules. We determined whether reads originated from one or both Xs in each cell. Dots represent SNP positions from *XIST* RNA split by XaXa-like, XaXi, and unknown cells on the x-axis. Dots are colored by broad category. Y-axis represents how much of each SNP came from one X or both Xs. In XaXi cells, 84.6% of *XIST* variants come from only one X. (f) Single X variants of *XIST* plotted in tiles by sample (broad grey boxes) and genomic position (x-axis). Single-allelic major (blue tiles) refers to the most abundant genotype at that position. Single-allelic minor (magenta tiles) refers to the alternative to single-allelic major. Mixed (yellow) refers to positions where some cells have alleles from both genotypes. (g) Genes plotted in ranked order by Spearman correlation to the X/A ratio. Highlighted are the top and bottom ten X-linked genes, highlighting that there are X-linked genes that strongly positively and negatively correlate with X/A ratio. Colors are the adjusted p-values ranging from 0 in red to 0.01 in blue; grey values are not considered significantly correlated. (h) Tree plot of semantically simplified gene set terms for top 20 gene ontology terms by adjusted p-value. Circle sizes are the number of genes in the term and colored by adjusted p-value.

Supplementary Tables *(in Excel)*

Supplementary Data 1. Orthogonal naming methods results for independent SCT samples

(a) Naming results based on ACT automated cell annotation via a hierarchically organized marker gene map (b) Results of naming by correlated clusters with published ovarian (column "CellName_OvTe") and human embryonic stem cell (ESC)-derived teratoma (column "CellName_CellDerived") datasets (c) Names by using Seurat's RunAzimuth, running the "fetusref" from Cao et al. (2020) and retaining the top ("Percentage") average score ("AverageScore") for each cluster. (d) Naming using a large language model-based framework where the top 50 genes from each cluster were provided to ChatGPT's gpt-4o model.

Supplementary Data 2. Marker genes for each cluster.

Markers for all 26 cell types in SCTs using the Wilcoxon Rank Sum test via the FindAllMarkers function (logfc.threshold = 0.25, only.pos = TRUE).

Supplementary Data 3. Principle component analysis genes and gene set results.

(a) Gene loadings that define principle component 1 ranked by highest to lowest contributions. (b) Gene set enrichment analyses results using gene ontology for PC1 ranked genes by loading contribution. (c) Gene loadings that define principle component 2 ranked by highest to lowest contributions. (d) Gene set enrichment analyses results using gene ontology for PC2 ranked genes by loading contribution.

Supplementary Data 4. Results of X-to-Autosomal ratio profiling using Spearman correlations and gene set enrichment.

(a) Gene correlations to X-to-Autosomal Ratio using Spearman correlation and to account for multiple comparisons, p-values were adjusted using the Benjamini-Hochberg FDR correction (qvalue) (b) Gene set enrichment analyses results using gene ontology for all genes (autosomal and X-linked) (c) Gene set enrichment analyses results using gene ontology using only the autosomal genes.
